# A Mobile App for Detecting Potato Crop Diseases

**DOI:** 10.3390/jimaging10020047

**Published:** 2024-02-13

**Authors:** Dunia Pineda Medina, Ileana Miranda Cabrera, Rolisbel Alfonso de la Cruz, Lizandra Guerra Arzuaga, Sandra Cuello Portal, Monica Bianchini

**Affiliations:** 1 Centro Nacional de Sanidad Agropecuaria, San José de las Lajas 11300, Cuba; dkpineda88@gmail.com (D.P.M.); ileanam@censa.edu.cu (I.M.C.); roly@censa.edu.cu (R.A.d.l.C.); lizguerra@censa.edu.cu (L.G.A.); sandra@censa.edu.cu (S.C.P.); 2 Dipartimento di Ingegneria dell’Informazione e Scienze Matematiche, Università degli Studi di Siena, 53100 Siena, Italy

**Keywords:** deep neural networks, image processing, classification of potato crop diseases

## Abstract

Artificial intelligence techniques are now widely used in various agricultural applications, including the detection of devastating diseases such as late blight (*Phytophthora infestans*) and early blight (*Alternaria solani*) affecting potato (*Solanum tuberorsum* L.) crops. In this paper, we present a mobile application for detecting potato crop diseases based on deep neural networks. The images were taken from the PlantVillage dataset with a batch of 1000 images for each of the three identified classes (healthy, early blight-diseased, late blight-diseased). An exploratory analysis of the architectures used for early and late blight diagnosis in potatoes was performed, achieving an accuracy of 98.7%, with MobileNetv2. Based on the results obtained, an offline mobile application was developed, supported on devices with Android 4.1 or later, also featuring an information section on the 27 diseases affecting potato crops and a gallery of symptoms. For future work, segmentation techniques will be used to highlight the damaged region in the potato leaf by evaluating its extent and possibly identifying different types of diseases affecting the same plant.

## 1. Introduction

The automatic detection of pathogens in plants, as early as possible and without damaging the plant, is an approach that is enjoying increasing success in the agrifood sector. In automatic detection, the basic assumption is that a diseased plant looks different from a healthy one. For example, leaves can exhibit subtle color differences, often invisible to the human eye but which can be captured using techniques such as spectral imaging. The detection of potato crop diseases is complicated because parasites and their eggs are often found under the canopy of plants and are therefore difficult to detect. They are often very small and show a very local distribution. Crops, in general, could be affected by multiple diseases at the same time. Therefore, not only high-resolution detection but also local and organism-specific detection is needed. High-resolution imaging, combined with deep learning (DL) techniques, especially convolutional neural networks (CNNs), could have the potential for precision agriculture for standard and greenhouse crops. In both cases, large quantities of labeled images from different situations (locations, seasons, crop varieties) are needed to sufficiently train deep learning algorithms. Moreover, augmentation and smarter training techniques are necessary to overcome the lack of real data and labeled images. Finally, transfer learning has also proven useful for the identification and diagnosis of diseases in agricultural crops, without ignoring the multiplicity of applications it has [1].

Particularly, potato (*Solanum tuberosum* L.) crops are constantly affected by the incidence of parasites which cause a decrease in their yield every year. Being a widespread crop in the world, the control of its production requires attention, and the problem of automatic disease recognition from leaf images via CNNs has been the subject of much recent literature, such as [2,3,4,5,6,7,8], to cite only some contributions. For instance, in [9], potato tuber diseases were diagnosed using the VGG architecture, by adding new dropout layers to avoid overfitting. As a result, 96% of the test images were classified correctly. After comparing MobileNet, VGG16, InceptionResNetV2, InceptionV3, ResNet50,VGG19 and Xception architectures, in [10], it was found that VGG16 had the highest accuracy (99.43%) on test data for the diagnosis of late blight and early blight, the most incident diseases in potato crops. Finally, in [11], a novel hybrid deep learning model, called PLDPNet, has been proposed, for automatic segmentation and classification of potato leaf diseases. The PLDPNet utilizes autosegmentation and deep feature ensemble fusion modules to enhance disease prediction accuracy, with an end-to-end performance of 98.66% on the PlantVillage dataset ([12], https://www.kaggle.com/datasets/emmarex/plantdisease, accessed on 12 December 2023).

The versatility of CNNs allows for their implementation on different platforms, including mobile devices. Mobile applications achieved rapid popularity because, in addition to being practical and lightweight, they simplify access to information and promote their widespread use. Their ecosystem is made up of several factors: infrastructure, operating system (OS), information distribution channels, etc. Nowadays, almost everyone owns a smartphone, whether it is an Android, iOS or other operating system device. Despite their diffusion, in Cuba, a large part of the population can only afford phones with low performance (Android version over 4.0, 2G data network with a population coverage of 85%, internal memory 1 GB, etc.). The quality–price ratio is an obstacle to technological updating, and therefore, obtaining a practical and lightweight tool for the identification of potato diseases is a necessary strategy to help farmers in controlling their crops.

The objective of this paper is to release an offline mobile application with the most effective machine learning architecture for the diagnosis of fungal blight in potatoes. The mobile application developed is compatible with Android versions higher than 4.1, has a storage capacity of 77.57 MB and does not require an Internet connection or mobile coverage. Other similar proposals can be found in the literature, as in [13], where a mobile app based on the MobileNetv2 architecture was developed, which can classify five disease categories: general early blight, severe early blight, severe late blight, severe late blight fungus and general late blight fungus. The model achieved an accuracy of 97.73%. Nonetheless, this study, as well as [14,15,16], requires high-resolution images and/or advanced features in the technological infrastructure, incompatible with the characteristics of Cuban mobiles, which are mostly on the way to obsolescence and unable to take even medium-quality pictures. In [17], a mobile application called VegeCare was devised for the diagnosis of diseases in potatoes, yielding 96% accuracy. However, it is a proprietary software, difficult to access for the Cuban community. Moreover, most of these studies propose an architecture that requires connection to an external server for image processing, as in [15]. While it is true that this works without extenuating circumstances in many countries, due to the availability of resources and access to free online platforms, it must be considered that in Cuba there are still planting regions where there is no mobile coverage or very low signal, which would hinder access to available international solutions.

Many mobile apps for smart agriculture have recently been devised based on deep learning [18], sometimes founded on proprietary software. However, these apps, in addition to not being free of charge, can only be installed on devices with current Android versions and, normally, refer to a client–server architecture where the information is stored in external databases. Therefore, they require mobile networks and an external server with a MySQL manager for queries [15]. In Cuba, the company GeoCuba has focused its efforts on image processing in the agricultural sector, mainly for the control of sugar cane and rice cultivation. Using satellite photos, drones and AI techniques, damages in these crops can be identified; however, this requires advanced tools to capture images in real time and platforms with high computational performance, not to mention that the distance at which the images are taken may hinder the efficiency of the diagnosis.

All the above motivations push towards having a simple mobile app that, in addition of being free, offline and suitable for the characteristics of devices with low performance, can also cover the role of a decision assistant. The real-time diagnosis of the main diseases contributes to the reduction in the risk of crop losses, to the early identification of the type of parasite and to the reduction in the use of pesticides, and therefore to ecological sustainability. It includes an important strategic component as it is an informative tool that helps non-expert personnel to know about different diseases present in the crops, produced by insects, viruses, bacteria and nematodes, as each one has degenerative factors on a medium or large scale in potato cultivation.

Summing up, the main contributions of this paper can be described as follows.

- We carried out an experimental study to find the best DL model for classifying potato diseases, based on leaf images, which represents a good compromise between computational lightness and performance;
- We have implemented the free PCD (Potato Crop Diseases) app, to help farmers detect potato crop diseases early, if equipped with a basic mobile phone.

The rest of the paper is organized as detailed below. In the following section, the PlantVillage dataset and the experimental setting are presented. In Section 3, our experimental results are reported, assessing the superiority of the MobelNetv2 architecture to be included in a mobile app for potato disease detection, while a brief discussion on similar studies carried out in the literature constitutes Section 4. The PCD app is briefly described in the subsequent Section 5. Finally, Section 6 traces some conclusions and future perspectives.

## 2. Materials and Methods

### 2.1. The PlantVillage Dataset

Potato crop leaf images were used as a case study, with a focus on the most incident diseases, late blight and early blight, identifying three classes by including healthy leaves. The *Phytophthora infestans* (late blight) fungus is a polycyclic disease, and in its various strains is responsible for both tomato and potato downy mildew, one of the most well-known and feared phytopathologies, favored by very prolonged rainfall followed by considerable air humidity with night-time dew. The Phytophtora symptoms are evident on the leaves—with young leaves more susceptible to infection—where necrotic light-colored spots appear that rapidly turn to dark brown and tend to dry out and affect the leaf surface first and then the entire aerial part of the plant and the tuber. Since late blight develops rapidly at moderate temperature and high humidity, a condition that corresponds to the climate situation in Cuba, where the rainy, humid and hot season goes from April to November, entire crops can collapse in less than a week [19]. The *Alternaria solani* (early blight) fungus forms very similar lesions on both leaves and stems and can affect the entire plant. On the leaves, the symptoms initially manifest themselves with green or light-brown spots presenting concentric rings which, as the disease progresses, transform into dark-brown angular lesions with a yellow halo limited by the leaf veins. Lesions can rupture easily. On the fruits, dark, sunken and leathery notches appear on the side of the stem. The early blight disease is preserved on crop residues of infected plants and spreads through rainwater or irrigation. As for late blight, the conditions predisposing the development of the pathogen occur with temperatures between 22 and 33 °C and high humidity levels [19].

**Figure 1:**
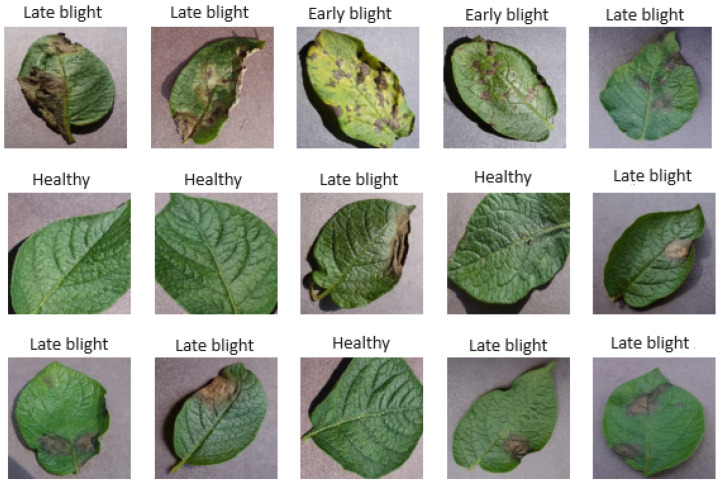
Sample images, taken from the PlantVillage dataset, corresponding to late blight, early blight and healthy potato leaves.

In recent years, the frequency of early and late blight has increased, probably also following the occurrence of predisposing climatic conditions (related to the general rise in temperatures). In both diseases, the main control measure suggested is to correctly identify the problem through early detection and classification. The data used in this study are extracted from the PlantVillage dataset (https://www.kaggle.com/datasets/emmarex/plantdisease, accessed on 12 December 2023). An average of 1000 images were used for each class, with resolution 96 × 96 pixels per inch, dimensions 256 × 256 pixels, and 24 bits in depth (see Figure 1). For each class, 700 images were used for training, 200 for validation and 100 for model evaluation.

### 2.2. Experimental Setting

The experiments were carried out with a Windows 11 Pro operating system, on a ×64 processor, Intel(R) Core(TM) i5-7200U CPU@2.50 GHz 2.71 GHz and with 8 GB RAM. For image processing, we made use of the Anaconda Navigator platform (v.2.1.4) and the Spyder IDE (v.5.1.5), employing TensorFlow (v.2.10.1), Keras (v.1.1.2), Matplotlib (v.3.7.1) and NumPy (v.1.23.4) libraries. Python and the Python interpreter (v.3.9) were used for the software implementation. The Android Studio development environment, the Kotlin programming language and TensorFlow-lite dependencies were used to develop the mobile application.

Five widely used CNNs have been evaluated, namely MobilNetv2 [20], VGG16 [21], VGG19 [22], InceptionV3 [23] and Xception [24], calculating the accuracy of each model, to select the best performing one. For each of the analyzed architectures, after a grid search procedure, the hyperparameters were set as listed below (with values in common for the first iterations), in order to make a fair comparison between models.

- Number of epochs: 10, 50;
- Activation function for the output layer: softmax;
- Optimizer: Adam;
- Loss function: sparse categorical cross–entropy;
- Batch size: 32, 50;
- Metrics: Accuracy.

The first considered model was MobileNetv2, which is a lightweight architecture particularly tailored to mobile applications, whose computational cost and processing time are significantly lower than the rest of the architectures tested in our experiments. It is based on an inverted residual structure and, as a whole, contains the initial full convolutional layer with 32 filters, followed by 19 residual bottleneck layers. ReLU6 is used as the activation function because of its robustness when used with low–precision hardware. Finally, the network has an image input size of 224 × 224 [20]. Instead, the VGG16 model has 13 convolutional layers followed by 13 fully connected layers—with ReLU activation—only 16 of which have learnable weights (hence the name) [21]. The network has an image input size of 224 × 224. VGG19 shares the same structure as VGG16, with the addition of three convolutional layers; thus, it has 19 trainable layers [22]. Inceptionv3 has a 42-layer architecture and processes images of size 229 × 229. It is computationally less expensive with respect to previous Inception architectures (v1 and v2) and can easily be retrained for custom image classification problems [23]. Finally, Xception is an extension of the Inception model, which uses the standard Inception modules with depth-separable convolutions. The Xception architecture has 36 convolutional layers forming the feature extraction base of the network. The 36 convolutional layers are structured into 14 modules, which have linear residual connections, except for the first and last modules. In short, the Xception architecture is a linear stack of depthwise separable convolutional layers with residual connections. The convolutional and separable convolutional layers are followed by batch normalization [24]. All experiments described in the following were based on pretrained architectures (CNN models presented in this section are saved in Github and are freely downloadable at https://github.com/dkpineda88/TransferLearninPapas.git (accessed on 12 December 2023)), fine-tuned on the PlantVillage dataset. Three steps were carried out:

- Step 1: Firstly, the models were trained for ten epochs;
- Step 2: Then, three new layers were added: (i) a dropout layer with a rate of 0.3 to avoid overfitting, (ii) a dense layer with ReLU activation functions and (iii) a softmax activation function in the output layer;
- Step 3: Finally, the new incorporated layers were kept (with a dropout rate of 0.5) and the number of epochs was increased to 50.

The three steps were performed considering both a batch size of 32 and 50 samples. In this work, the trained models were converted into tflite files for optimization and processing in the Android Studio platform. Finally, a model deployment module was built to store the trained neural networks in the Kotlin framework (for this purpose, the following dependencies were installed: ‘org.tensorflow:tensorflow-lite:2.4.0’, ‘org.tensorflow:tensorflow-lite-support:0.1.0’, ‘org.tensorflow:tensorflow-lite-metadata:0.1.0’, ‘org.tensorflow:tensorflow-lite-gpu:2.3.0’).

For the validation of the application, specialists and technicians from the Plant Health Directorate of the Cuban Centro Nacional de Sanidad Agropecuaria (CENSA) were selected. Subsequently, a survey was applied to measure the degree of acceptance of the system and its effectiveness in diagnostics. The survey included nine questions to be evaluated on a scale of one to five. For the evaluation of late blight and early blight classification, the users employed their own images extracted from field samples taken by experts from the Plant Health Directorate of CENSA.

## 3. Results

The increase in the cost of energy and raw materials in Cuba is causing a new concept in agricultural production techniques, and the use of IT tools, mainly based on artificial intelligence, paves the way for developments capable of revolutionizing agricultural work. The goal of smart agriculture is to increase profits and, of course, reduce the risks of capital loss and destruction of natural resources. Mobile applications for plant disease detection represent a smart strategy and their use is currently essential to strengthen food sustainability, especially given the lack of investment in infrastructure plaguing the Cuban agricultural system. To obtain a CNN model capable of significantly reducing computational costs, being adaptable to the performance of mobile devices and capable of processing images effectively, it is necessary to take into account several incident factors (e.g., the number of model parameters and processing times), mainly related to the limited computational resources available. In fact, traditional deep learning models cannot be applied directly to mobile devices.

Therefore, after investigating lightweight neural network architectures and using transfer learning to limit the computational load due to training, the MobileNetv2 architecture was found to have the best adaptability to the data, with the highest level of accuracy, the lowest number of parameters and the lowest number of epochs (see Table 1).

**Figure 2:**
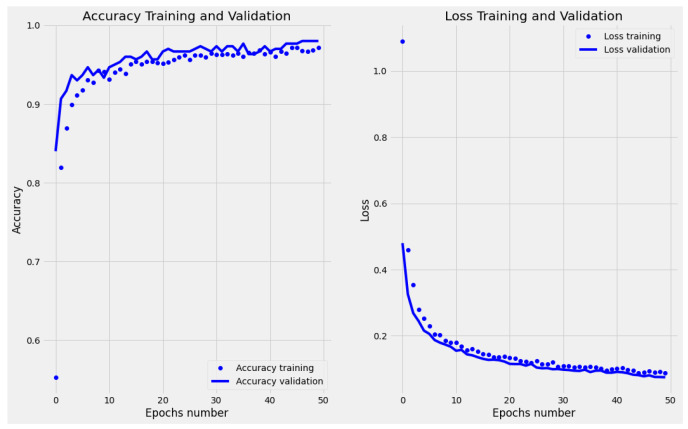
MobileNetv2 accuracy and loss on the training and validation data, respectively (number of epochs on the *x* axis).

**Figure 3:**
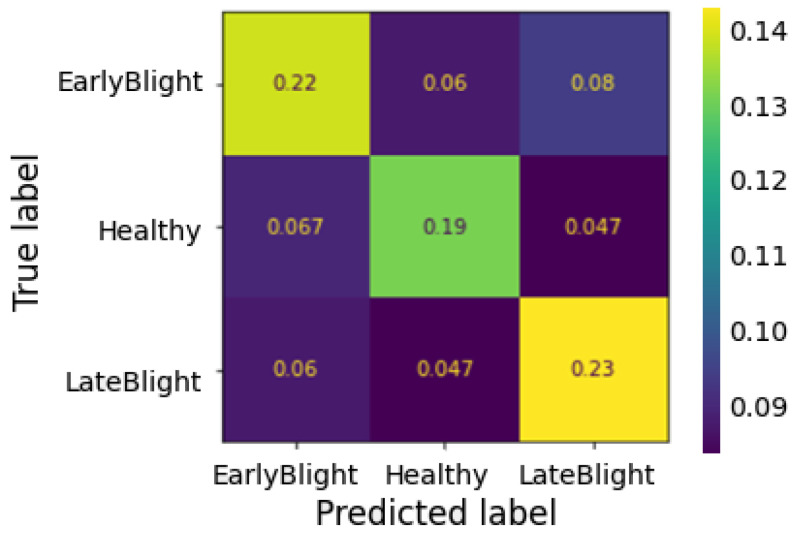
MobileNetv2 results on the test set summarized in the confusion matrix.

**Figure 4:**
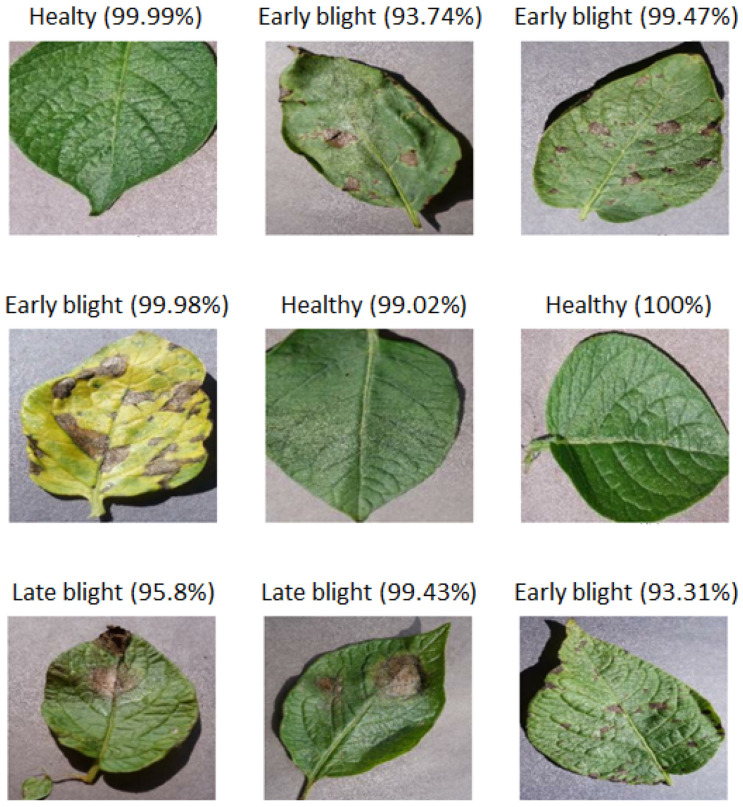
MobileNetv2 correct prediction on some test data. The confidence (in brackets) with which the network takes its decision is reported for each sample image.

After training the MobileNetv2 only for ten epochs, data overfitting was observed, both for a batch size of 32 and 50. Instead, adding the layers described in Step 2, the accuracy on the validation set shows that the model is slightly underfitted. Finally, executing Step 3, the validation set accuracy remains relatively aligned with that on the training data (see Figure 2). The confusion matrix shows a more specific than sensitive model (see Figure 3); however, the misclassifications of the diseased leaves, out of the total number of test samples, represent less than 5% (Figure 4). Finally, considering our problem in a binary classification framework, Precision and Recall show high and balanced values (≈0.83 and 0.84, respectively), which indicates an unbiased classifier in recognizing healthy and diseased leaves, and therefore the ability to both find all relevant pathological cases while identifying them selectively.

## 4. Discussion

The results obtained in our experiments differ from those reported in [6] where, after applying ten deep learning models such as DenseNet201, DenseNet121 [25] (a densely connected convolutional network (DenseNet) is a feedforward architecture in which each layer is linked to every other layer. This allows the network to learn more effectively by reusing features, hence reducing the number of parameters and enhancing the gradient flow during training. The number attached to the name stands for the number of layers), NASNetLarge [26] (a neural architecture search network (NASNet), from Google Brain, utilizes reinforcement learning with a recurrent neural network-based controller to search for an efficient building block for a small dataset (CIFAR10), which is then transferred to a larger dataset (ImageNet), by stacking multiple copies of the building block. NASNetLarge owes its name to the higher resolution of the images it is able to process, namely 331×331 pixels (relative to other NAS models)), Xception, ResNet152v2 [27] (residual networks (ResNet) use skip connections. Skip connections distribute activations of a layer to further layers by skipping some layers in between. This forms a residual block. ResNets are made by stacking residual blocks together. By using skip connections, alternative paths are provided for the gradient (with backpropagation). These additional paths are beneficial for the model convergence. The improvement in ResNetv2 is mainly found in the arrangement of layers—batch normalization-ReLU and convolutions exchanged—in the residual block. The number attached to the name stands for the number of layers), EfficientNetB5, EfficientNetB7 [28] (in the EfficientNet architecture, a scaling method is used to uniformly scale all dimensions of depth, width and resolution using a compound coefficient. The baseline network of EfficientNet was built with a NASNet incorporating squeeze and excitation in the building block of MobileNetV2. Greater numbers stand for larger models able to process higher-resolution images), VGG19 and MobileNetv2, along with the hybrid model EfficientNetB7–ResNet152v2 for classification, it resulted that DenseNet201 obtained the highest accuracy, equal to 98.67%, with a validation error of 0.04. However, the model covers not only potato but also tomato and bell pepper diseases, with a total of 15 classes. Instead, in [5], the VGG16 model was selected, achieving 100% accuracy on the test data, after also evaluating VGG19, MobileNetv2, Inceptionv3 and Resnet50v2.

Generally speaking we can state that the results (accuracy) obtained in the present work conform to the performance of the other DL methods present in the literature which, as described both in Section 1 and in the previous discussion, present a performance that vary approximately from 96% to 100%, with the best accuracy obtained in the case of more complex architectures (see also https://paperswithcode.com/sota/image-classification-on-plantvillage (accessed on 12 December 2023) for the related leaderboard). Nevertheless, neither network size nor processing speed were taken into account in those studies, although they are necessary elements for a model to be encapsulated in a mobile app, which is the ultimate goal of the present research.

## 5. Deployment of the PCD Mobile App

Mobile devices have limited storage and computation capability, mostly due to the battery consumption, a constraint that should be considered when deploying a DL–based app. Indeed, there are two different ways that a DL inference can be performed with a mobile phone, based on a cloud platform or on device. Cloud-based deep learning, also called Edge-DL, is carried out using cloud-exposed APIs that host a pretrained model. Conversely, the on-device approach presupposes the use of mobile CPUs and GPUs to run the DL software and the phone memory to store the model [29]. A new trend is to use custom hardware and/or co-processors to accelerate machine/deep learning applications, but such hardware devices are present on new processors only with which just high-end cell phones are equipped [30]. Instead, this paper focuses on low-power devices without hardware acceleration support, which represents 2/3 of the current market. Nonetheless, the advantages of the on-device approach lies in being able to work without Internet access, guaranteeing data privacy and having no cloud hosting costs.

**Figure 5:**
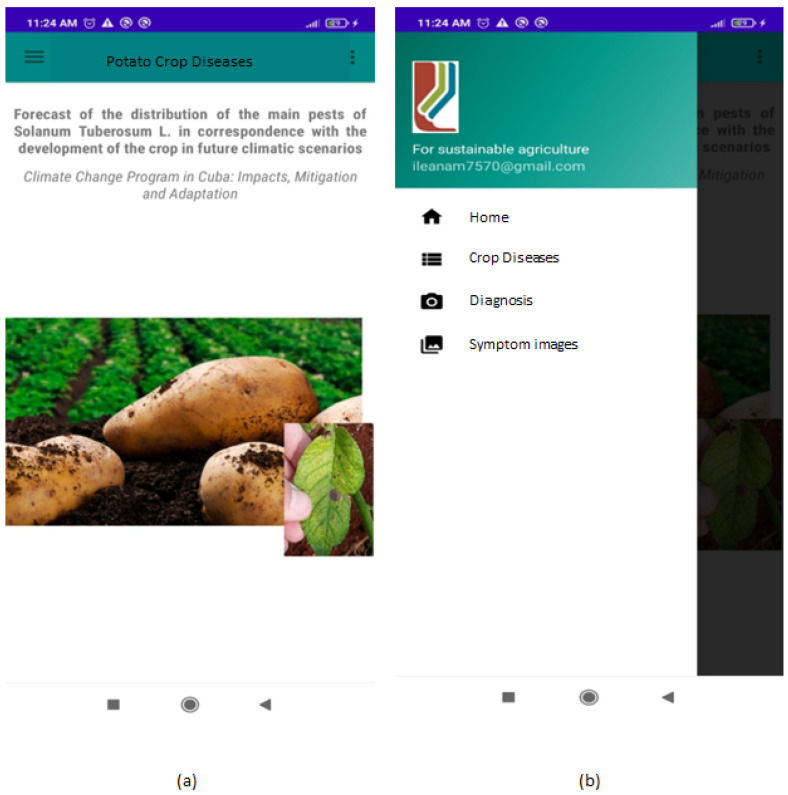
Graphic interface of the PCD app; main page (**a**) and side menu (**b**).

In 2022, there were 7.6 million mobile cellular subscriptions in Cuba, which translated into a penetration rate of approximately 68% (as for the STATISTA Research Department, https://www.statista.com/aboutus/our-research-commitment, accessed on 12 December 2023), with an estimated increase to 71.1 during 2023. However, obtaining internet access can still be a tricky process for some Cubans, at least those living in rural areas, whereas Cubacel, the Cuban company that provides cell phone service throughout the island, covers all the main cities and tourist destinations in the country. Furthermore, rural people can often only afford low-performance phones. For these reasons, the PCD mobile app is compatible with Android version higher than 4.1, requires 77.57 MB of storage and does not require Internet connection or mobile coverage. The interface of the application presents a brief description of the project (see Figure 5a) and a side menu with the following options: (i) Home (link to the main page), (ii) Crop diseases, (iii) Diagnosis, and (iv) Symptom images (Figure 5b).

**Figure 6:**
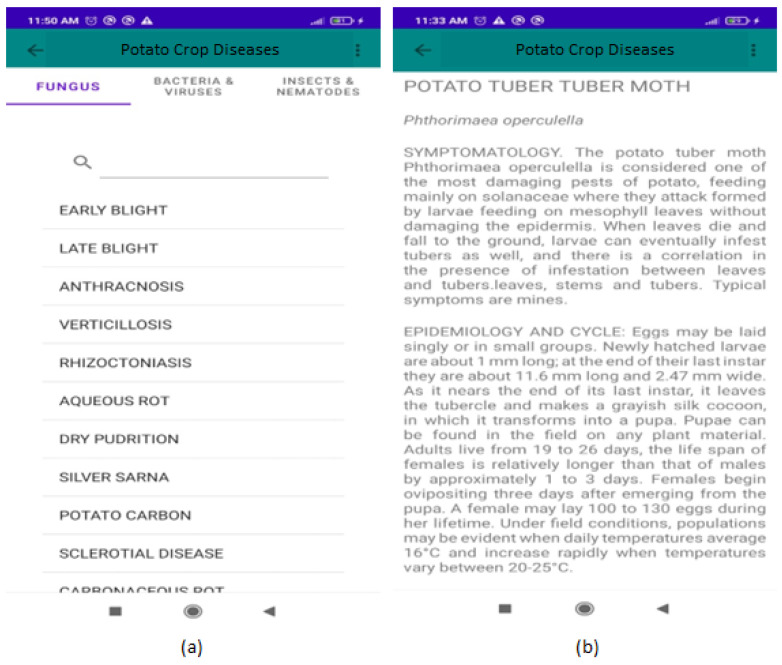
Graphic interface of the PCD app for the list of diseases (**a**) and the detailed description of a specific disease (**b**).

Option (ii) has the objective of showing the 27 diseases that can affect potato crops, and at the same time briefly describes their main characteristics: scientific name, symptomatology, epidemiology and cycle and control techniques. This section shows the list of diseases subdivided by the causal agents (Fungi, Bacteria and Viruses, Insects and Nematodes, Figure 6a). Clicking on the disease of interest, its description will be displayed (Figure 6b).

**Figure 7:**
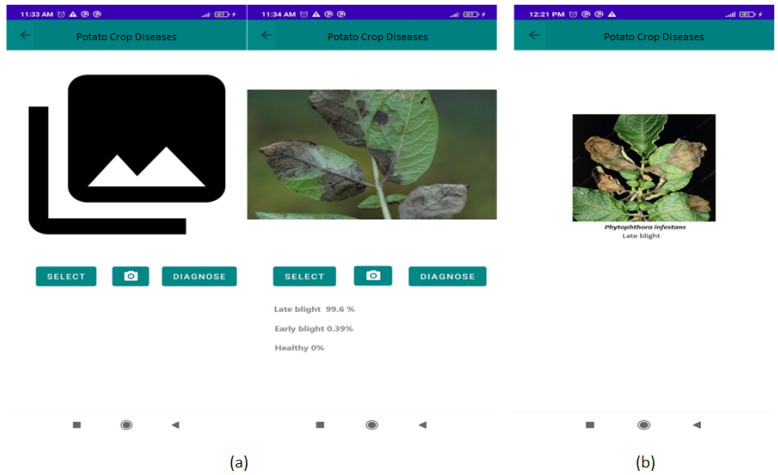
Graphic interface of the PCD app for the diagnosis of late blight and early blight given an image (**a**); a diseased leaf example (**b**).

Option (iii) responds to the main objective of this work, as it is responsible for diagnosing, through an image, the percentage of presence of late blight, early blight or establishing that the plant is healthy (Figure 7a). Images can be selected from the gallery of the mobile device or can be taken in real time in the field. Finally, option (iv) allows the user to visualize, through images, the behavior of the symptoms according to the diseases described in option (ii) (Figure 7b).

The mobile application was developed with the objective of providing technicians or specialists in crop evaluation with an affordable tool for the timely diagnosis of the most devastating diseases that affect potato crops. The user survey yielded an acceptance rating of 4.3 out of 5 points, a result that demonstrates its usefulness in decision making and the manageability of accessing this resource.

## 6. Conclusions

In this paper, we have proposed an experimental study on different deep network architectures in order to find the most suitable to be used in a mobile app for potato disease identification. A major constraint was that of choosing a lightweight model to be used on obsolete hardware/software mobile phones in Cuba that are also unable to access the network. Preliminary experimental results are promising. Future work will be devoted to preventively apply some segmentation techniques on the leaf images to diagnose not only the type of disease but also its severity (namely the extension of the leaf surface interested by the presence of stains), which is important especially when leaves may be affected by more than one disease. Moreover, other diseases—such as rhizottoniosis, caused by the fungus *Rhizoctonia solani* and bacteriosis caused by the strain *Erwinia carotovora*—which produce alterations to the leaves and the exposed part of the plant (as well as to the tuber) will be included among the pathologies that can be classified via the PCD app. Finally, enriching the image collection by the final users—who can capture pictures with the cellular phone in real conditions—will be valuable, especially in view of a future where cloud computing will be an option also in Cuba.

## Figures and Tables

**Table 1:** Hyperparameter setting and best results for the five CNN models.

CNN Type	Step ^1^	# Trained Param.	# Frozen Param.	# Epochs	Accuracy	Loss	Model Size
MobileNetv2	3	81,984	2,257,984	50	0.987	0.0623	3.89 MB
VGG16	2	1539	14,714,688	10	0.94	0.38	14.2 MB
VGG19	1	1539	20,024,384	10	0.9844	0.0415	19.2 MB
Inceptionv3	1	153,603	21,802,784	10	0.91	5.7269	21.4 MB
Xception	3	6147	20,861,480	50	0.9467	0.1394	21.1 MB

^1^ The Step column defines at which of the above described training steps the network reaches its best performance.

## Data Availability

Data are contained within the article.
